# Relationship of an *hRAD54* gene polymorphism (2290 C/T) in an Ecuadorian population with chronic myelogenous leukemia

**DOI:** 10.1590/S1415-47572010005000095

**Published:** 2010-12-01

**Authors:** César Paz-y-Miño, Andrés López-Cortés, María José Muñoz, Bernardo Castro, Alejandro Cabrera, María Eugenia Sánchez

**Affiliations:** 1 Instituto de Investigaciones Biomédicas, Facultad de Ciencias de la Salud, Universidad de las Américas, QuitoEcuador; 2 Escuela de Ciencias Biológicas, Facultad de Ciencias Exactas y Naturales, Pontificia Universidad Católica del Ecuador, QuitoEcuador

**Keywords:** cancer, leukemia, CML, ALL, *hRAD54*, 2290 C/T polymorphism

## Abstract

The *hRAD54* gene is a key member of the *RAD52* epistasis group involved in repair of double-strand breaks (DSB) by homologous recombination (HR). Thus, alterations of the normal function of these genes could generate genetic instability, shifting the normal process of the cell cycle, leading the cells to develop into cancer. In this work we analyzed exon 18 of the *hRAD54* gene, which has been previously reported by our group to carry a silent polymorphism, 2290 C/T (Ala730Ala), associated to meningiomas. We performed a PCR-SSCP method to detect the polymorphism in 239 samples including leukemia and normal control population. The results revealed that the 2290 C/T polymorphism has frequencies of 0.1 for the leukemia and 0.1 for the control group. These frequencies show no statistical differences. Additionally, we dissected the leukemia group in chronic myelogenous leukemia (CML) and acute lymphoblastic leukemia (ALL) to evaluate the polymorphism. The frequencies found in these subgroups were 0.14 for CML and 0.05 for ALL. We found statistically significant differences between CML patients and the control group (p < 0.05) but we did not find significant differences between ALL and the control group (p > 0.05). These results suggest a possible link between the 2290 C/T polymorphism of the *hRAD54* gene and CML.

DNA repair is a major feature for maintenance of genome stability and integrity in living cells. DNA double-strand breaks (DSB) are lesions that threaten the integrity of the genome. If not properly processed, DSB can lead to cell cycle arrest or illegitimate DNA rearrangements such as translocations, inversions, or deletions, which contribute to cell dysfunction, cell death, or carcinogenesis (Wesoly *et al.*, 2006).

It is known that the chromosomal aberrations that activate oncogenes and trigger the carcinogenic process always involve double-stranded DNA exchanges. This phenomenon may be produced by DSB (Lengauer *et al.*, 1998). One major repair pathway in eukaryotic cells in order to eliminate deleterious effects is homologous recombination (HR) (Kooistra *et al.*, 1997). HR is generally a precise way of resolving DSB and successful chromosome segregation during meiosis (Mazina and Mazin, 2008). Moreover, dysregulation of HR may lead to aberrant genetic rearrangements and genomic instability, resulting in various mutations (Park *et al.*, 2008).

The *hRAD54* gene plays an important role in homologous recombination and DNA double-strand break repair. Rad54 belongs to the Snf2/Swi2 protein family. It possesses a robust DNA-dependent ATPase activity, uses ATP hydrolysis to supercoil DNA and cooperates with the Rad51 recombinase in DNA joint formation (Smirnova *et al.*, 2004; Rossi and Mazin, 2008). This gene has been mapped at chromosome 1p32 by fluorescent *in situ* hybridization (FISH) (Kanaar *et al.*, 1996). The (1p32) region has been found to be a site of high rates of loss of heterozygosity, and concentrates six single nucleotide polymorphisms (SNP) that have been associated with genetic instability in meningiomas (Leone *et al.*, 1999, 2003). *hRAD54* also has been proposed as an oncosupressor in breast cancer and several point mutations in conserved regions of the *hRAD54* gene have been found in primary tumors such as colonic adenocarcinoma, non-Hodgkin lymphoma and breast carcinoma (Smirnova *et al.*, 2004). Moreover, the 1p32 region is a common partner for balanced translocation in hematological and lymphoid malignancies, *e.g.* t(1;14) (p32;q11) and t(1;3) (p32;q27) in leukemia and lymphoma, respectively (Cotter, 1998; Rubnitz and Pui, 1998).

In this work, we analyzed the status of the 2290 C/T silent polymorphism in exon 18 of the *hRAD54* gene in the Ecuadorian population. It is reported that the polymorphism is associated with meningiomas (Leone *et al.*, 2003), suggesting that this alteration could be involved in tumorigenesis. Therefore, the objective of this study was to relate the T allele distribution to leukemia development.

Leukemia is a malignant disease which is characterized by the presence of carcinogen cells in bone marrow and blood. According to the cancer cell type involved, this disease is classified as lymphocytic and myelogenous, which are divided in turn into acute and chronic. Therefore, leukemia is divided into four categories: acute lymphoblastic leukemia (ALL), chronic lymphoblastic leukemia (CLL), acute myelogenous leukemia (AML) and chronic myelogenous leukemia (CML) (Gabert *et al.*, 2003).

239 Ecuadorian individuals were analyzed, 137 individuals with clinical diagnosis of different types of leukemia (ALL = 61, CLL = 1, AML = 15, CML = 60) and 102 healthy individuals with no leukemia diagnosis as a normal control population. In Ecuador the most important ethnic group is known as “mestizo” (~60% of the total Ecuadorian population), the result of the mixture of Amerindians and Spaniards (Paz-y-Miño, 1998). The study was approved by the Ethical Committee of the Pontifical Catholic University of Ecuador. All study subjects received counseling and provided written consent for the study.

DNA extraction from bone marrow (leukemia patients and control population) was performed as described by (Sambrook *et al.*, 1989). The bone marrow samples obtained from healthy individuals were taken prior to other medical diagnoses. The *hRAD54* gene polymorphism 2290 C/T, in exon 18, was determined by the polymerase chain reaction - single-strand conformation polymorphism (PCR-SSCP) method. PCR was performed in a MJ Research PTC-200 thermal cycler (MJ Research, Watertown, Mass., USA) using primers designed by (Rasio *et al.*, 1997). The amplification reaction was carried in a final volume of 20 μL, containing 1 X reaction buffer, 2.5 mM MgCl_2_ (Invitrogen, Carlsbad, CA), 200 μM of each dNTP, 2 μM of each sense and antisense primers, 1 U of *Taq* polymerase (Invitrogen, Carlsbad, CA) and 100 ng of genomic DNA as template. PCR conditions consisted of an initial denaturation at 94 °C for 5 min; followed by 35 cycles of 94 °C for 30 s, 54 °C for 1 min, 1 min 30 s at 74 °C, and a final extension at 72 °C for 8 min. The amplified fragment of 242 bp was then denatured at 91 °C for 5 min and snap-cooled for SSCP detection by electrophoresis on a 12% non-denaturing polyacrylamide (49:1 acrylamide:bisacrylamide) gel with 10% of glycerol.

Statistical analyses were done using PASW Statistics 17 for Windows (SPSS, Chicago). Allelic and genotypic frequencies were also calculated. The non-parametric Fisher's exact test was applied to determine significant differences between affected (CML = 60, ALL = 61) and healthy individuals.

The PCR-SSCP method allowed detection of the three genotypes of the *hRAD54* gene polymorphism 2290 C/T (C/C, C/T, T/T) within the studied populations. From the total 239 individuals analyzed (478 alleles in total), 199 (83.26%) were homozygous for the normal allele (C/C), 38 (15.9%) had a heterozygous genotype (C/T) and two individuals (0.84%) were homozygous for the polymorphism (T/T). Of the 42 T alleles present in the studied population, 27 (64.29%) belonged to the study group and 15 (35.71%) to the control group. In the study group 17 (62.96%) individuals with CML and 6 (22.22%) individuals with ALL, presented the T allele (the percentages were calculated from the base of 27 alleles present in the total study group). The details of the genotypic distributions and allele frequencies of each group are presented in Table 1. The statistical analysis showed no significant differences between the control population and the study group (p > 0.05). When we applied the statistical test over the CML and ALL subgroups we found differences in the CML subgroup (p < 0.05), whereas the ALL showed no differences (p > 0.05).

HR is required to maintain genomic stability and its absence leads to potentially oncogenic translocations and other karyotypic changes. While this pathway exists to effect accurate and “safe” repair, it also has the potential to misrepair and generate deleterious products. Consequently, there is strong evolutionary pressure for mechanisms of apoptosis and cellular senescence which favor eliminating a cell with chromosomal damage from a dividing population over the risk of errant repair and oncogenic transformation (Ferguson and Frederick, 2001).

We found Hardy-Weinberg equilibrium in the allele frequency of the study population. Polymorphisms in genes that are responsible for maintenance of genome stability, such as *hRAD54*, could increase the risk to acquire cancer (Liu *et al.*, 1999; Paz-y-Miño *et al.*, 2003).

When the frequency of the polymorphism in the leukemia group (0.1) was compared with that in the normal control population (0.1), no statistical difference (p > 0.05) was observed. However, when we divided the study group into its two main subgroups, CML and ALL, the calculated frequencies for the CML subgroup (0.14) showed statistically significant differences (p < 0.05) from the control population, whereas those calculates for the ALL subgroup (0,05) did not. In spite of the results obtained from the statistical tests, it is important to note that size of the sample is not large; and that this may pose a limitation for the interpretation of the results.

It has been reported that t(9;22), responsible for some types of leukemia, especially CML, may be the result of an aberrant HR process, because the BCR and ABL genes involved in this translocation share some homology in their sequences (Bishop and Schiestl, 2002). These findings suggest that the recombinational repair of DSB by HR or spontaneous HR are both carried out by the *RAD52* epistasis group (Mazina and Mazin, 2004); among which group the Rad54 protein plays an especially important role. The 2290 C/T polymorphism of the *hRAD54* gene could be part of the mutator phenotype responsible for the appearance and progression of malignant transformation (Paz-y-Miño *et al.*, 2003). However, this mutator phenotype seems to be linked to CML, but not to other types of leukemia (Sellick *et al.*, 2008), suggesting that in fact the translocation involved in CML, t(9;22), is the result of mistakes of the DSB repair pathway by HR, in which the *hRAD54* gene plays a key role (Bishop and Schiestl, 2002; Symington, 2002).

## Figures and Tables

**Table 1:** Distribution of the 2290 C/T polymorphism in leukemia (CML, ALL) and control populations.

Group	Genotype	Individual (%)	Genotypic frequency	Allele frequency
\=tbody Leukemia (*n* = 137)	C/C	112 (82)	0.82	0.9
	C/T	23 (17)	0.17	
	T/T	2 (1)	0.01	0.1
CML (*n* = 60)	C/C	45 (75)	0.75	0.86
	C/T	13 (22)	0.22	
	T/T	2 (3)	0.3	0.14
ALL (*n* = 61)	C/C	55 (90)	0.90	0.95
	C/T	6 (10)	0.10	
	T/T	0 (0)	0	0.05
Control (*n* = 102)	C/C	87 (85)	0.85	0.925
	C/T	15 (15)	0.15	
	T/T	0 (0)	0	0.075
